# Inhibitory Activity of Eleven *Artemisia* Species from Iran against *Leishmania Major* Parasites

**Published:** 2012

**Authors:** Seyed Ahmad Emami, Shahrzad Zamanai Taghizadeh Rabe, Ali Ahi, Mahmoud Mahmoudi

**Affiliations:** 1 * Department of Pharmacognosy, School of Pharmacy, Mashhad Universityof MedicalSciences, Mashhad, Iran*; 2 * Immunology Research Centre, BuAli Research Institute, Mashhad University of Medical Sciences, Mashhad, Iran*; 3 * Immunology Research Centre, BuAli Research Institute, School of Medicine, Mashhad University of Medical Sciences, Mashhad, Iran*

**Keywords:** Artemisia, Leishmanicidal activity, Leishmania major, MTT assay, Promastigote

## Abstract

**Objective(s):**

Annual incidence of cutaneous leishmaniasis is increasingly growing and development of the alternative drugs against it is a major concern. *Artemisia* genus is a traditional medicinal plant in Iran. The aim of this study was to examine the leishmanicidal activity of various Iranian *Artemisia* species extracts.

**Materials and Methods:**

Different extracts were gathered from eleven Iranian *Artemisia* species. Their leishmanicidal activities against the growth of *Leishmania major *(*L. major*) promastigotes were examined as the half maximal inhibitory concentration (IC_50_) using MTT assay.

**Results:**

Obtained results showed that ethanol extracts especially those taken from *A. ciniformis*, *A. santolina* and *A. kulbadica* have the strongest effects.

**Conclusion:**

Looking for the effective leishmanicidal agents from natural resources in Iran, we found that the ethanol extract of collected *Artemisia* species had significant effect on *in vitro* leishmanicidal activity and may be suitable candidates in the treatment of leishmaniasis.

## Introduction

The genus *Artemisia* L. (Astraceae) is a large, heterogeneous and widely distributed genus throughout the world. These species are perennial, biennial and annual herbs or small shrubs. The genus *Artemisia* L. has 30 species in Iran out of which two are endemic (1). *Artemisia* plants contain chemical compositions such as monoterpenes, sesquiterpenes, sesquiterpene lactones, flavonoides, coumarins, sterols and polyacetylenes (1). Some of the biological activities of different *Artemisia* species include cytotoxic (2, 3, 4) and anti-inflammatory (5) activity. 

*Leishmania major (L. major*) is the protozoan parasite responsible for cutaneous leishmaniasis with annual incidence rate of 1.5 million people throughout the world. According to the World Health Organization (WHO) report, 12 million people are infected by parasites and 350 million people are living in regions with high risk of infection (6). Pentavalent antimonial drugs are prescribed as the first-line drugs for the treatment of leishmaniasis, but they have unpleasant side-effects and sometimes are toxic and non-effective. Resistance towards these medicines has been observed (7). In Mashhad (northeast of Iran), the incidence of cutaneous leishmaniasis is increasingly growing (6). Various medicinal plants have been used for treatment of leishmaniasis in this area. 

Previously, leishmanicidal activity of the extracts and natural products of some *Artemisia* species was reported (8-13). However, leishmanicidal activity of some other species of *Artemisia *has not been previously evaluated. In this study we prepared different extracts of eleven *Artemisia* species from Khorasan province, Iran and examined their leishmanicidal activity against *L. major* promastigotes* in vitro*. 

## Materials and Methods

Eleven species of *Artemisia* were collected from different areas of Iran. Their Persian names are shown in Table 1. Dr V Mozaffarian, Research Institute of Forest and Rangelands, Ministry of Jahad Keshavarzi, Iran, confirmed the identity of these plants. 

The shade dried aerial parts of each species (100 g) were chopped in small pieces and then crushed into powder by a blinder. Each sample was macerated in pure methanol for 24 hrs. The samples then were extracted using a percolator. The extracted solutions were concentrated at 50 °C under reduced pressure to dryness. Adequate amount of water was added to EtOH to obtain a 95%-aqueous methanol solution. This solution was added to the concentrated extract and then extracted with an equal volume of hexane. The methanol layer was evaporated to dryness and then was suspended for a second time in water. The suspension was partitioned between CH_2_Cl_2_ and EtOAc, successively. Each obtained extract was concentrated at 50 C under reduced pressure to dryness. 

*L. major* strain MRHO/IR/75/ER promastigotes were kindly provided by Dr Sazegarnia (BuAli Research Institute) cultured in RPMI 1640 supplemented with 10% heat-inactivated fetal calf serum, 2 mM *L-glutamine* and penicillin-streptomycin at 27 °C, in an atmosphere of 5% CO_2_ in an incubator.

To determine IC_50_ values, the 3-(4, 5-dimethylthiazol-2-thiazolyl)-2, 5-diphenyl-2H-tetrazolium bromide (MTT) method was used (8-13). Briefly, *L. major* promastigotes were seeded at 4×10^5^/well in 96-well micro plates under appropriate culture conditions. Then, different concentrations (10-2000 μg/ml) of *Artemisia* extracts were added and incubated at 27 °C in 5% CO_2_ for 24 hrs. After incubation, 10 μl of MTT solution (10 mg/ml) was added to each well followed by incubation for another 4 hrs. The enzyme reaction was then stopped by addition of 100 μl of 50% isopropanol–10% sodium dodecyl sulfate. Two or more independent experiments in triplicate were performed for the determination of sensitivity to each extract. As a control, the activity of *Artemisia* extracts in the absence of *L. major* promastigotes was also determined. 

**Table 1:** Characteristics of collected Artemisia samples from different parts of Iran

*Artemisia *species	Persian name	Location	Collection date
*A. annua *L.	Gandwash	Islamabad near Maraveh tapeh-Shahrabad road, North Khorasan province, northeast of Iran, height 940 m	15 Sep. 2003
*A. biennis* Willd.	Dermaneh Dosaleh	Near of Chovailly-Bajgiran road Ghuchan, Razavi Khorasan province, east of Iran, height 1650 m	24 Dec. 2004
*A. ciniformis *Krasch. & Popov ex Poljakov	Dermaneh Talaee	Islamabad near Maraveh tapeh-Shahrabad road, North Khorasan province, northeast of Iran, height 940 m	8 Aug. 2003
*A. sieberi *Besser	Dermaneh Dashti	Ghorogh Samie abad, Torbatjam, North Khorasan province, east of Iran, height 909 m	15 Sep. 2003
*A. kulbadica *Boiss. & Buhse	Dermaneh Kulbadi	Islamabad near Maravehtapeh-Shahrabad road, North Khorasan province, northeast of Iran, height 940 m	24 Dec. 2004
*A. santolina *Schrenk	Dermaneh Sefid	Between Khosph-Birjand, Birjand, South Khorasan province, east of Iran, height 1290 m	19 Sep. 2003
*A. turanica *Krasch.	Dermaneh Kermez	Ghorogh Samie abad, Torbatjam, Razavi Khorasan province, east of Iran, height 909 m	15 Sep. 2003
*A. absinthium* L.	Afsantin	Islamabad near Maraveh tapeh-Shahrabad road, North Khorasan province, northeast of Iran, height 940 m	15 Sep. 2003
*A. fragrans *Willd.	Dermaneh Moatar	Islamabad near Maraveh tapeh-Shahrabad road, North Khorasan province, northeast of Iran, height 940 m	23 Dec. 2004
*A. khorassanica *Podl.	Dermaneh Khorasani	Near Chovailly-Bajgiran road, Ghuchan, Razavi Khorasan province, east of Iran, height 1650 m	23 Dec. 2004
*A. kopedaghensis* Krasch., M. Pop. & Lincz. ex Poljak	Dermaneh Kopetdaghi	Near Bazangan lake, Sarakhs, Razavi Khorasan province, east of Iran, height 1030 m	8 Aug. 2003

The optical density (OD) at 570 nm was measured using an ELISA plate reader (Convergys EL-Reader, Convergent Technologies, Germany). The inhibitory concentration (IC_50_) of different extracts was evaluated graphically by plotting concentration versus percentage growth inhibition. 

## Results

The inhibitory concentration (IC_50_) of all tested extracts was determined using MTT method (Table 2). Extracts of all the eleven *Artemisia* species showed leishmanicidal activity. Although, all tested extracts exhibited antileishmanial activity after 24 hrs of incubation, but ethanol extracts of *A.kulbadica* (IC_50_: 25 μg/ml), *A. ciniformis* (IC_50_: 25 μg/ml) and *A. santolina *(IC_50_: 80 μg/ml) had the most potent leishmanicidal activity. 

**Table 2:** Leishmanicidal activities of *Artemisia* species on promastigote forms of *Leishmania major*

Scientific name	Mean of IC_50_ for extracts (μg/ml)			
	Ethanol	Ethyl acetate	Dichloromethane	Hexane
*A. turanica*	200 ± 1.3	675 ± 2.1	425 ± 0.9	1120 ± 2.5
*A. annua*	400 ± 0.8	425 ± 1.5	850 ± 0.9	1900 ± 2.4
*A. absinthium*	500 ± 0.6	425 ± 1.3	600 ± 0.8	1050 ± 2.5
*A. fragrans*	1000 ± 2.0	1375 ± 2.2	475 ± 1.0	1150 ± 2.2
*A. kulbadica*	25 ± 0.5	275 ± 1.4	440 ± 0.7	885 ± 1.8
*A. ciniformis*	25 ± 0.4	340 ± 1.2	450 ± 1.0	790 ± 1.7
*A. santolina*	80 ± 0.8	375 ± 1.1	675 ± 1.4	850 ± 1.4
*A. khorassanica*	400 ± 1.1	435 ± 0.7	500 ± 1.2	790 ± 1.5
*A. kopedaghensis*	50 ± 0.7	255 ± 0.8	445 ± 0.5	925 ± 1.6
*A. sieberi*	150 ± 1.0	265 ±0.7	465 ± 0.8	850 ± 1.5
*A. biennis*	100 ± 0.9	425 ± 0.5	525 ± 1.1	1050 ± 2.0

## Discussion

Current estimates show that leishmaniasis affected 88 countries and about 350 million people are exposed to the risk of contracting this disease [WHO (World Health Organization), Communicable disease surveillance and response]. The cutaneous leishmaniasis is also a major health problem in Mashhad (North-East of Iran). Limited efficacy, resistance to the drug, high cost and toxic side effects are the main drawbacks of available drugs against leishmaniasis. Medicinal plants have long been used for the treatment of different diseases and ailments such as cutaneous leishmaniasis without any scientific explanation for the mechanism of action of such preparations (6, 7). 

In the present study, we demonstrated the inhibitory effect of different extracts from eleven *Artemisia *species on the growth of *L. major *promastigotes *in vitro*. 

It was previously reported that the aqueous extract and essential oil of *A. herba alba* have antileishmanial activity against *L. tropica* and *L. major* promastigotes (8). Also, the aqueous extract of leaves of *A. indica* exhibited leishmanicidal activity (IC_50_: 430 μg/ml) (9). Here, some of tested *Artemisia* spp showed the most strong antileishmanial activity. 

In this study, all tested extracts exhibited antileishmanial activity after incubation, however ethanol extracts from *A. kulbadica* and *A. ciniformis* showed the most potent leishmanicidal activity (IC_50_: 25 μg/ml). Growth inhibitory activities of ethanol extract from other plants such as *Haplophyllum myrtifolium* Boiss. (IC_50_: 10.9 μg/ml) against *L. tropica* promastigotes were previously reported (10). 

Comparing the antilaishmanial effect of non-polar extracts revealed that ethyl acetate extract of *A. fragrans* had less antileishmanial activity against *L. major* promastigotes. Ethyl acetate extracts of studied *Artemisia* species (except for *A. turanica* and *A. fragrans*) were also more active in comparison with their dichloromethane extract. *In vitro* antileishmanial activity of ethylacetate and dichloromethane extracts of *Ircinia spinosula* (IC_50_: 16.09, 47.38 μg/ml) was reported against *L. major* promastigotes (11). The LD_50_ of dichloromethane extract and hexane extract of *Calophyllum brasiliense *on *L. amazonensis* promastigotes was respectively 40 mg/ml and 20 mg/ml (12).

In comparison with other extracts, studied *Artemisia* species hexane extracts (except for *A. fragrans*), were less active against *L. major*. Hexane extracts of *A. annua*, *A. fragrans*, *A. turanica*, *A. absinthium* and *A. biennis* were less effective than other species. Other investigators have also reported lower activity of hexane extracts of plants against *leishmania* species in comparison with other extracts. For example, ethanol but not hexane extracts of *Arbutus unedo* significantly decreased *L. tropica* promastigotes counts (13). 

Although phytochemical screening of different *Artemisia* species has shown the presence of monoterpenes, sesquiterpenes, sesquiterpene lactones, flavonoides, coumarins, sterols and polyacetylenes (1, 14), but there is little information about their leishmanicidal effect. In this study, we assumed the leishmanicidal activity of tested extracts to the presence of these classes of natural compound(s). In conclusion, we found that the ethanol extracts of most of *Artemisia* species had favorable leishmanicidal activity and kill *L. major *promastigotes in a dose-dependent manner. Further fractionation of these *Artemisia* species and isolation of their compounds is required to pinpoint their antileishmanial constituents.

## Conclusions

Looking for the effective leishmanicidal agents from natural resources in Iran, we have found that the ethanol extract of collected *Artemisia* species showed powerful *in vitro* leishmanicidal activity and may be suitable candidates in the treatment of leishmaniasis.
